# Identification of ferroptosis-related diagnostic markers in primary Sjögren's syndrome based on machine learning

**DOI:** 10.4317/medoral.26190

**Published:** 2023-10-12

**Authors:** Huimin Yang, Chao Sun, Xin Wang, Tao Wang, Changhao Xie, Zhijun Li

**Affiliations:** 1 Graduate School, Jinan University, Guangzhou, China; 2 Department of Rheumatology and Immunology, the Second Affiliated Hospital of Bengbu Medical College, Anhui; 3 Key Laboratory of Infection and Immunity of Anhui Higher Education Institutes, Bengbu Medical College, Anhui; 4 Department of Rheumatology and Immunology, the First Affiliated Hospital of Bengbu Medical College, Anhui

## Abstract

**Background:**

Primary Sjogren's syndrome (pSS) is a common autoimmune disorder that affects up to 0.3-3% of the global population. Ferroptosis has recently been identified to play a significant role in autoimmune diseases. However, the molecular mechanisms of ferroptosis in the initiation and progression of pSS remains unclear.

**Material and Methods:**

To investigate the molecular mechanisms underlying the occurrence and progression of pSS, we utilized a comprehensive approach by integrating data obtained from the Gene Expression Omnibus (GEO) database with data from the FerrDb database to identify the ferroptosis-related differentially expressed genes (DEGs). Furthermore, we implemented an innovative transcriptomic analysis method utilizing a computer-aided algorithm to establish a network between hub genes associated with ferroptosis and the immune microenvironment in pSS patients.

**Results:**

Our results revealed significant differences in the gene expression profiles of pSS samples compared to normal tissues, with 1,830 significantly up-regulated genes and 1,310 significantly down-regulated genes. In addition, our results showed a significant increase in the proportions of B cells and CD4+ T cells in pSS samples compared to normal tissues. AND then, our analysis revealed that a combination of six ferroptosis-related genes, including TBK1, SLC1A4, PIK3CA, ENO3, EGR1, and ATG5, could serve as optimal markers for the diagnosis of pSS. The combined analysis of these six genes accurately diagnosed the occurrence of pSS.

**Conclusions:**

This study offers valuable insights into the pathogenesis of pSS and highlights the importance of targeting ferroptosis-related DEGs, which suggests a novel treatment strategy for pSS.

** Key words:**Primary Sjögren's syndrome, ferroptosis, diagnostic markers, machine learning.

## Introduction

Primary Sjogren's syndrome (pSS) is a common autoimmune disorder that affects up to 0.3-3% of the global population (1). pSS is characterized mainly by keratoconjunctivitis sicca and xerostomia, which result from the glandular infiltration of immune cells, particularly in the exocrine glands. Patients with pSS may also experience musculoskeletal pain and fatigue, which significantly impair their quality of life (2). Furthermore, up to one-third of pSS patients may develop severe systemic manifestations, including tubulointerstitial nephritis, neuropathy, interstitial pneumonia, or hemocytopenia, which can negatively impact their prognosis (3). The development of an autoimmune response in pSS patients is thought to result from a combination of genetic, environmental, and infectious factors. However, the exact mechanisms behind these events leading to pSS remain unknown. Given the lack of effective treatment, identifying early diagnostic markers, and gaining a better understanding of the pathogenesis of pSS is essential for the timely intervention with pSS patients.

Ferroptosis is an iron-dependent form of regulated cell death (RCD) that has recently been identified to play a significant role in the onset and progression of various diseases, including autoimmune diseases (4). The accumulation of lipid peroxides and iron-dependent reactive oxygen species (ROS) are characteristic features of ferroptosis, with iron metabolism and lipid peroxidation signaling acting as crucial mediators of this process (5). Extensive research has examined the pathological role of ferroptosis in autoimmune diseases such as systemic lupus erythematosus (SLE), rheumatoid arthritis (RA), inflammatory bowel diseases (IBD), and multiple sclerosis (MS) (6). For instance, studies have shown that GPX4 plays a critical role in the occurrence and progression of SLE. Autoantibodies and interferon-α present in the serum induce neutrophil ferroptosis through enhanced binding of the transcriptional repressor CREMα to the GPX4 promoter. This leads to the suppression of GPX4 expression and subsequent elevation of lipid-reactive oxygen species (7). In this context, understanding the implications of ferroptosis in relation to pSS could lead to the development of effective treatments for patients with this autoimmune disorder. However, further investigations are needed to determine the exact role of ferroptosis in the pathophysiology of pSS.

With the rapid development of molecular biology, numerous studies have been conducted on the pathophysiology of pSS, which identified several biomarkers beneficial in the diagnosis and treatment (3,8). However, despite the significant advances made in pSS research, the role of ferroptosis in the initiation and progression of the disease remains unclear. The current literature suggests that the pathogenesis of pSS is related to the intricate cellular communication between immune cells and epithelial cells, leading to immune system dysregulation (9). Particularly, various studies showed that T cells play critical roles in the pathophysiology of pSS, which are known to involve in various processes (10). The overactivation of B cells was also demonstrated to contribute to the exacerbation of pSS symptoms (11). Although the immune system plays a crucial role in the pathogenesis of pSS, the dominated cells which are involved in pSS via the process of ferroptosis remains unknown. Further research is required to investigate the potential role of ferroptosis in the mechanisms underlying pSS progression. Identification of ferroptosis-related molecular mechanisms could provide a foundation for developing more targeted and effective therapies for patients with pSS.

In the current study, we utilized a comprehensive approach by integrating data obtained from the Gene Expression Omnibus (GEO) database with data from the FerrDb database to identify the differentially expressed genes (DEGs) that are involved in ferroptosis. Furthermore, we implemented an innovative transcriptomic analysis method utilizing a computer-aided algorithm to establish a network between hub genes associated with ferroptosis and the immune microenvironment in pSS patients. Our study provides a comprehensive analysis of ferroptosis pathway in pSS and offers novel insights into potential therapeutic strategies.

## Material and Methods

- Data acquirement and preprocessing

Gene expression data sets GSE127952, GSE154926, GSE159574, GSE40611, and GSE97614, were obtained from the GEO database (https://www.ncbi.nlm.nih.gov/geo/). GSE40611, GSE97614, and GSE127952 were microarray data sets that contained 17 pSS and 18 control parotid tissue samples, 9 pSS and 3 control parotid tissue samples, and 8 pSS and 6 control small salivary glands, respectively. GSE154926 and GSE159574 were high-throughput sequencing counting datasets that contained 43 pSS and 7 control secondary salivary glands, and 16 pSS and 13 non-pSS salivary glands, respectively. We used the inter-array normalization function in the R software package “limma” (Version 3.56.1) to remove the batch effect differences between these data sets. We also employed the maximum value method to remove duplicate gene names corresponding to multiple probes. After removing batch effect differences, we obtained a total of 93 pSS samples and 47 non-pSS samples. Principal component analysis (PCA) was performed using the R software packages “FactoMineR” (Version 2.8) and “factoextra” (Version 1.0.7). Visualization of the results was achieved using the R software package “ggplot2” (Version 3.4.0).

- Differential gene expression analysis

To perform gene differential expression analysis, we utilized the R software package “limma” (Version 3.56.1). Only genes with a P-value less than 0.05 and a log2 fold change greater than 0.5 or less than -0.5 were selected as differentially expressed genes (DEGs). The DEGs were further analyzed using the R software packages “EnhancedVolcano” (Version 1.18.0) and “ggheatmap” (Version 2.2) to generate volcano maps and heat maps, respectively. We utilized the Ferroptosis database (http://www.zhounan.org/ferrdb/current/) to obtain a list of 568 ferroptosis-related genes, which included drivers, suppressors, markers, and inducers of ferroptosis. To identify ferroptosis-related differential gene sets, we compared the DEGs with the ferroptosis-related gene list obtained from the Ferrdb database. The intersection of these two gene sets was visualized using a Venn diagram.

- Functional enrichment analysis

To analyze the cellular components (CC), biological processes (BP), and molecular functions (MF) of differentially expressed genes (DEGs), we conducted gene ontology (GO) enrichment analysis. Furthermore, we utilized gene set enrichment analysis (GSEA) to identify biologically related pathways that were significantly up-regulated in patients with primary Sjogren's syndrome (pSS). We also performed Kyoto Encyclopedia of Genes and Genomes (KEGG) pathway enrichment analysis to identify signaling pathways that were significantly altered in DEGs and ferroptosis-related DEGs. To conduct these analyses, we used the R software package “clusterProfiler” (Version 4.8.1) for annotation, visualization, and integrated discovery. P-value < 0.05 was considered statistically significant.

- PPI network and identify hub genes

We constructed a protein-protein interaction (PPI) network for ferroptosis-related differentially expressed genes (DEGs) using the STRING database (https://cn.string-db.org/). To identify hub genes in the ferroptosis-related DEGs, we utilized the “Mcode” algorithm. We visualized the biological networks and identified key DEGs using the Cytoscape software (http://www.cytoscape.org/).

- Screening of the optimal ferroptosis-related diagnostic markers

In order to identify the most effective diagnostic markers for pSS, a total of 154 samples were randomly allocated into training and test sets at a ratio of 7:3. The least absolute shrinkage and selection operator (LASSO) algorithm and random forest algorithm, implemented in the R software packages “glmnet” (Version 4.1-7) and “randomForest” (Version 4.7-1.1), respectively, were utilized to screen for diagnostic markers associated with ferroptosis. The resulting gene sets from both algorithms were combined and analyzed to determine the most optimal ferroptosis-related diagnostic markers. The performance of the diagnostic markers was evaluated using the receiver operating characteristic (ROC) curve, which was generated using the R software package ROCR (Version 1.0-11). A significance level of *P* < 0.05 was employed to determine statistical significance.

- Immune cell infiltration analysis

In order to assess the extent of immune cell infiltration in pSS tissues compared to normal samples, we employed the R software package “xCell” (https://github.com/dviraran/xCell) to estimate the proportion of 19 distinct immune cells present in different samples, based on their RNA expression levels. The resulting data on immune infiltration were then visualized using the R software packages “pheatmap” (Version 1.0.12) and “ggpubr” (Version 0.6.0).

- Correlation analysis between diagnostic markers and immune microenvironment

To further explore the relationship between ferroptosis-related diagnostic markers and immune infiltration, we conducted a correlation analysis between 6 markers and 19 types of immune cells using both Mantel test. The resulting correlations were then visualized using the R software packages “ggplot” (Version 3.4.0) and “ggcor” (https://github.com/hannet91/ggcor).

## Results

- Differential gene expression analysis in integrated pSS cohorts

**Figure 1:**
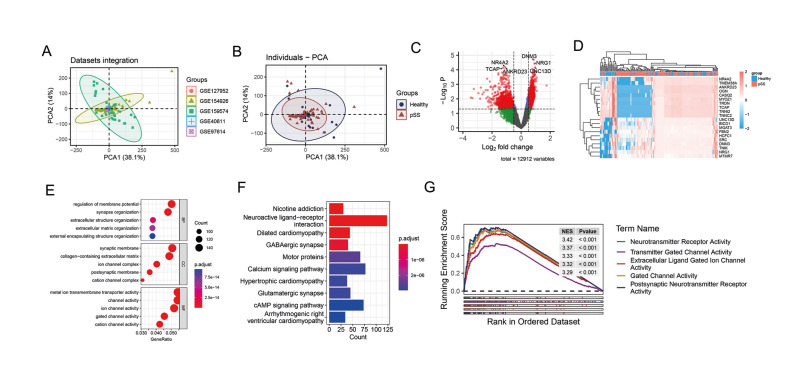
Differential gene expression analysis and functional enrichment analysis in integrated pSS cohorts. (A) Principal component analysis of five independent pSS patient cohorts from the GEO database. (B) Principal component analysis of the healthy and pSS samples. (C) Volcano plot of DEGs between the normal and pSS samples. (D) Heatmap of DEGs between the normal and pSS samples. (E) GO analysis results of DEGs between pSS patients and normal controls. (F) KEGG analysis results of DEGs between pSS patients and normal controls. (G) GSEA results of DEGs between pSS patients and normal controls.

To comprehensively investigate the molecular mechanisms underlying the occurrence and progression of pSS, we integrated five independent pSS patient cohorts from the GEO database using the limma package. Principal component analysis was conducted to evaluate the impact of batch effects on the samples during the integration process. The results demonstrated that the batch effect was significantly eliminated, and there were discernible differences in the principal components of the normal and pSS tissues (Fig. 1). To further explore the genes with significantly altered expression levels in pSS, differential gene analysis was performed using the limma package. The results, presented in the form of a volcano plot and heat map, revealed that 1830 genes, including DNM3, NEG1, and UNC13D, were significantly up-regulated, while 1310 genes, such as NR4A2, TCAP, and ANKRD23, were significantly down-regulated in pSS tissues compared to normal tissues (Fig. 1). These findings indicate that transcriptome expression changes exist between pSS patients and healthy individuals.

- Functional Enrichment Analysis in integrated pSS cohorts

To further investigate the potential biological functions of DEGs, we employed the R software package clusterProfiler to perform gene enrichment analysis based on the GO and KEGG databases. Additionally, we assessed the activation score of signaling pathways using GSEA. The GO enrichment analysis revealed that the regulation of membrane potential and extracellular structure organization were significantly enriched in the BP category, while the synaptic membrane and ion channel complex were significantly enriched in the CC category, and the metal ion transmembrane transporter activity and ion channel activity were significantly enriched in the MF category (Fig. 1). The KEGG enrichment analysis showed that the nicotine addiction and calcium signaling pathway were significantly enriched (Fig. 1). Furthermore, the GSEA results demonstrated that the neurotransmitter receptor activity and transmitter gated channel activity were significantly activated in pSS samples, with normalized enrichment scores (NES) of 3.42 and 3.37, respectively (Fig. 1).

- Identification of ferroptosis-related DEGs and Hub genes

**Figure 2:**
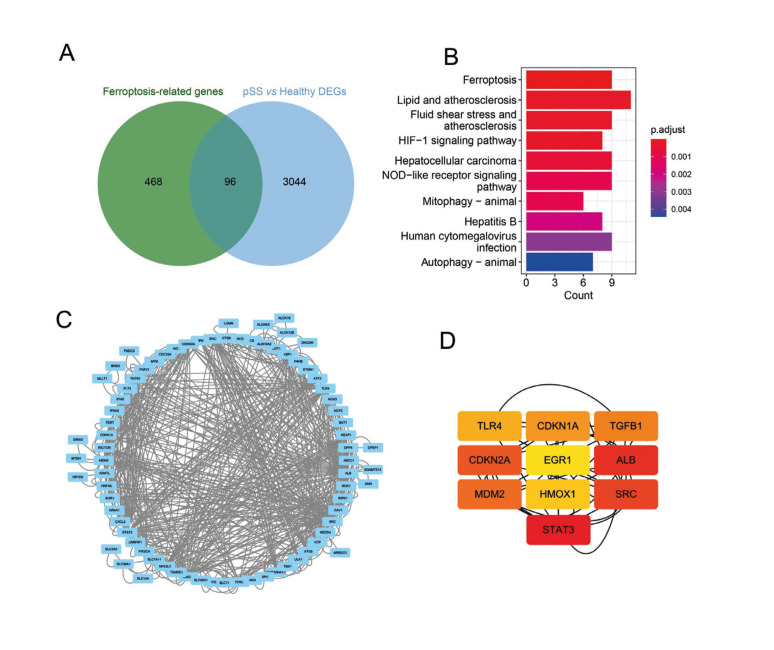
Identification of ferroptosis-related DEGs and Hub genes. (A) Venn diagrams showing the overlap between ferroptosis-related genes obtained from the Ferrdb database and significant DEGs from the control and pSS samples. (B) KEGG analysis results of ferroptosis-related DEGs between pSS patients and normal controls. (C) Construction of the PPI network based on ferroptosis-related DEGs. (D) Construction of hub genes networks using the Mcode algorithm in the Cytoscape software.

To investigate the changes of ferroptosis-related genes in pSS, we conducted a cross-analysis of 568 ferroptosis-related genes obtained from the Ferrdb database with the DEGs identified in pSS tissues. The results revealed that the expression of 96 genes related to ferroptosis was significantly altered in pSS tissues (Fig. 2). To further explore the potential signaling pathways involved in the regulation of ferroptosis-related DEGs, we performed KEGG pathway enrichment analysis. The results showed that the ferroptosis, lipid and atherosclerosis and HIF-1 signaling pathway were significantly enriched (Fig. 2). Additionally, we constructed a protein interaction network based on the STRING online database, which revealed potential interactions among these 96 genes (Fig. 2). Subsequently, we identified the hub genes of ferroptosis-related DEGs based on the Mcode algorithm. The results indicated that genes STAT3, SRC, ALB, and CDKN2A play key roles in the regulation of ferroptosis-related DEGs in pSS (Fig. 2).

- Identify ferroptosis-related diagnostic markers based on machine learning

**Figure 3:**
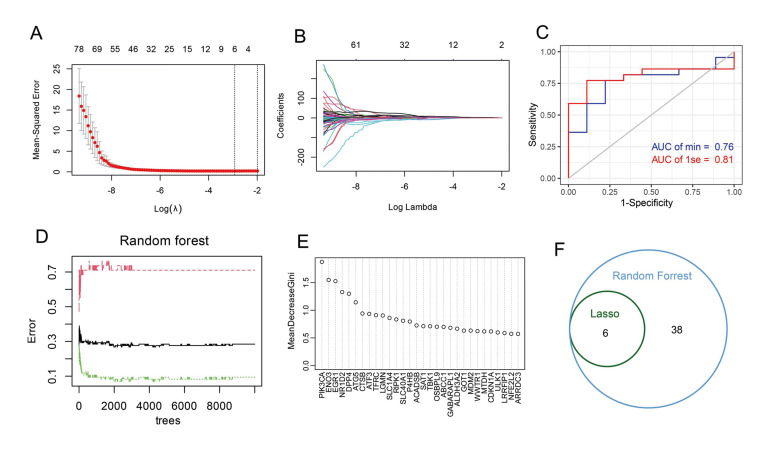
Identify ferroptosis-related diagnostic markers based on machine learning. (A-B) LASSO regression algorithm to screen the optimal ferroptosis-related DEGs. (C) The ROC curves of the six genes in the test set. (D) Random forest regression algorithm to screen the optimal ferroptosis-related DEGs. (E) Venn diagrams showing the overlap between optimal diagnostic markers identified from LASSO regression algorithm and random forest regression algorithm. (F) Venn diagram of key genes identified by the LASSO algorithm and the random forest algorithm.

To identify optimal diagnostic markers among the ferroptosis-related DEGs, we divided 154 patients into a training set and a test set using a 7:3 ratio, and employed the LASSO and random forest algorithms. The LASSO logistic regression algorithm identified six optimal genes, including TBK1, SLC1A4, PIK3CA, ENO3, EGR1, and ATG5, as prognostic markers in all ferroptosis-related DEGs (Fig. 3). The test set results demonstrated excellent specificity and sensitivity in the diagnosis of pSS patients, with an ROC > 0.75 (Fig. 3). Furthermore, the random forest algorithm identified 46 genes with Gini coefficients of more than 0.5 (Fig. 3). Notably, the cross-analysis results revealed that the six genes identified by LASSO remained effective in the random forest algorithm (Fig. 3). These findings suggest that the six genes identified by the LASSO algorithm are reliable diagnostic markers for pSS patients.

- Immune cell infiltration analysis in integrated pSS cohorts

**Figure 4:**
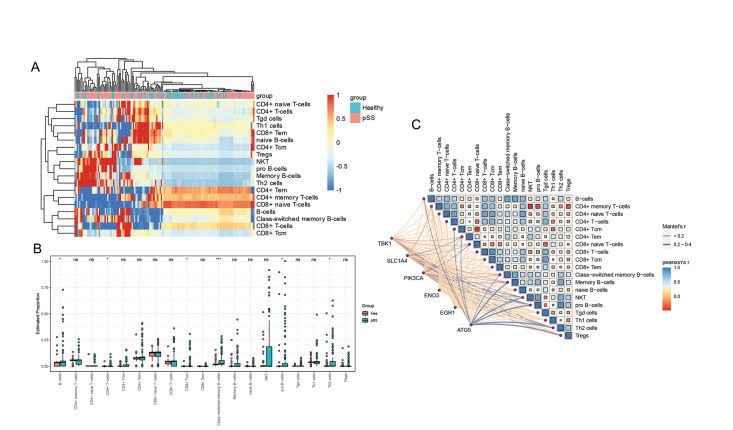
Immune cell infiltration analysis in integrated pSS cohorts. (A) Heatmap shows the proportions of 19 immune cells in pSS samples. Each column represents an individual patient sample, and each row represents an immune cell, ordered by unsupervised hierarchical clustering. (B) Boxplot of the estimated proportions of 19 immune cells based on single-sample gene set enrichment analysis (ssGSEA) in pSS and normal tissues. (C) Correlations between 6 ferroptosis-related diagnostic markers and the proportions of 19 immune cells. Color represents Pearson’s correlation coefficient r of each immune cells, with blue color indicating a positive correlation (Pearson’s r > 0), red color indicating a negative correlation (Pearson’s r < 0). Statistical analysis was done with the Mantel test, with blue line indicating *P* value <0.01, orange line indicating 0.01 < *P* < 0.05.

To investigate immune infiltration in pSS and normal tissues, we utilized the xCell algorithm to calculate the proportion of 19 types of immune cells in different samples (Fig. 4). The results revealed a significant change in the proportion of immune cell infiltration between pSS and normal tissues. Specifically, the proportion of B cells, CD4+ T cells, CD8+ Tcm, memory B cells and Th2 cells increased significantly in pSS tissues (Fig. 4). These findings suggest that the dysregulation of the immune microenvironment plays a crucial role in the pathogenesis and progression of pSS.

- Association between diagnostic markers and immune microenvironment

To further investigate the relationship between the six optimal ferroptosis-related diagnostic markers and the immune microenvironment in patients with pSS, we employed the Mantel test to calculate the Pearson correlation between ferroptosis-related diagnostic markers and 19 immune cells. The results revealed a significant correlation between ferroptosis-related diagnostic markers and immune cell infiltration. Specifically, the expression level of TBK1 was negatively correlated with the infiltration of CD4+ Tem and CD8+ T cells, while the expression level of ATG5 was positively correlated with the infiltration of memory B cells, NKT cells, Th2 cells, and Treg cells (Fig. 4). These findings suggest that ferroptosis-related diagnostic markers may regulate the pathogenesis and progression of pSS by modulating immune cell infiltration.

Discussions

pSS is a prevalent autoimmune disease that primarily affects middle-aged and elderly women (12). Recent advancements in the understanding of the pathogenesis of pSS have led to the identification of various biomarkers and potential therapeutic targets through molecular genetics and cell biology, including anti-Sjögren’s syndrome-related antigen A (anti-SSA) and anti-Sjögren’s syndrome-related antigen B (anti-SSB) antibodies (13). However, the complexity of the immune microenvironment and the diverse pathogenesis of pSS have hindered the complete elucidation of the molecular mechanisms and biomarkers involved (14). Thus, there is an urgent need to explore the pathogenesis and development mechanisms of pSS to uncover the underlying biology of the disease and develop effective comprehensive treatment strategies.

In this study, we aimed to investigate the gene expression profiles and signaling pathways associated with pSS by integrating gene expression data from multiple independent pSS cohorts based on public databases. We performed differential analysis and pathway enrichment analysis to identify genes and pathways with significant changes in pSS. Our results revealed significant differences in the gene expression profiles of pSS samples compared to normal samples, with 1,830 significantly up-regulated genes and 1,310 significantly down-regulated genes. Notably, we found that DNM3 and UNC13D were up-regulated, both of which have been reported to be associated with overactivation of the immune system (15,16), suggesting that pSS may progress due to immune system overactivation. Additionally, our enrichment analysis showed significant activation of calcium signaling pathway and extracellular matrix organization, which are consistent with previous reports (17,18). These findings provide valuable insights into the molecular mechanisms underlying pSS and may help to identify potential therapeutic targets.

The pathogenesis of autoimmune diseases is often associated with an imbalance in the immune microenvironment, including changes in the ratio of various immune cells (19). Therefore, we investigated the infiltration of immune cells based on the gene expression profile of pSS patients. Our results showed a significant increase in the proportions of B cells and CD4+ T cells in pSS samples compared to normal tissues, indicating that B cells and T cells play crucial roles in regulating lymphocyte infiltration and tissue destruction of exocrine glands in pSS. Although the etiology of pSS is not fully understood, numerous studies have reported that T cells and B cells are the primary sources of proinflammatory cytokine production and autoantibody secretion, promoting the development of pSS by producing various autoantibodies and inflammatory factors, including anti-nuclear antibody (ANA), anti-SSA, and anti-SSB (20,21). Our findings confirm the accuracy of the analytical method used in this study.

Ferroptosis is a form of cell death that occurs due to excessive accumulation of iron within cells (22). It is characterized by cell membrane damage, mitochondrial dysfunction, DNA damage, and cell apoptosis (23). In recent years, multiple studies have demonstrated that ferroptosis plays a critical role in various diseases, including malignancies, neurodegenerative disorders, atherosclerosis, and several autoimmune diseases (22). For instance, the histone methyltransferase G9a has been shown to regulate genes associated with ferroptosis, thereby promoting autoimmune encephalomyelitis and human multiple sclerosis by activating the ferroptosis pathway (24). Similarly, GPX4 overexpression in systemic lupus erythematosus nephritis promotes glomerular injury and lupus nephritis through ferroptosis pathways (25). However, the molecular mechanisms underlying ferroptosis in pSS have not been fully elucidated. Understanding the molecular mechanisms of ferroptosis in pSS is crucial for developing effective therapeutic strategies.

In this study, we aimed to identify ferroptosis-related genes that potentially contribute to the pathogenesis of pSS through joint analysis with differential genes. Our findings revealed that STAT3, ALB, and CDKN2A are potential ferroptosis-related genes involved in pSS. Phosphorylated STAT3 has been reported to contribute to the pathogenesis of pSS by regulating the differentiation of T follicular helper cells (26). Similarly, ALB has been shown to exacerbate pSS symptoms by regulating the immune microenvironment and may serve as a potent biomarker for the diagnosis of pSS (27). Furthermore, CDKN2A has been reported to mediate the progression of pSS by regulating senescence in salivary gland progenitor cells (28). These results support the notion that ferroptosis plays a crucial role in the occurrence and progression of pSS.

Furthermore, we utilized various machine learning algorithms to identify the optimal biomarkers associated with ferroptosis in pSS. Our analysis revealed that a combination of six genes, including TBK1, SLC1A4, PIK3CA, ENO3, EGR1, and ATG5, could serve as optimal markers for the diagnosis of pSS. The combined analysis of these six genes accurately diagnosed the occurrence of pSS. In addition, we investigated the correlation between these six genes and immune infiltration in pSS. Our results showed that these six genes were involved in the regulation of immune cell proportions in pSS. Notably, TBK1 and PIK3CA have been reported to play key roles in various autoimmune diseases and are potential therapeutic targets (29,30). Our study further demonstrated that TBK1 and PIK3CA regulate the occurrence and development of pSS through ferroptosis and immune infiltration pathways. These results provide important insights into the molecular mechanisms underlying pSS and highlight the potential of machine learning algorithms in identifying optimal biomarkers for the diagnosis of this autoimmune disease.

Overall, through an integrated approach, we have successfully identified numerous DEGs related to ferroptosis in pSS patients. By investigating the regulatory mechanisms of these DEGs within the immune microenvironment, we have gained a comprehensive understanding of the molecular mechanisms underlying ferroptosis in pSS. This novel perspective provides a solid foundation for further research in the field and holds great potential for the development of more effective diagnostic and therapeutic strategies for individuals with pSS. Our findings offer valuable insights into the pathogenesis of pSS and highlight the importance of targeting ferroptosis-related DEGs, which suggests a novel treatment strategy for pSS.

29/8/2023
